# Serosurvey on Household Contacts of Marburg Hemorrhagic Fever Patients

**DOI:** 10.3201/eid1203.050622

**Published:** 2006-03

**Authors:** Matthias Borchert, Sabue Mulangu, Robert Swanepoel, Modeste Lifenya Libande, Antoine Tshomba, Amayo Kulidri, Jean-Jacques Muyembe-Tamfum, Patrick Van der Stuyft

**Affiliations:** * Institute of Tropical Medicine, Antwerp, Belgium;; † Institut de Recherche Biomédicale, Kinshasa, Democratic Republic of Congo;; ‡ National Institute for Communicable Diseases, Johannesburg, South Africa;; § Ministry of Health, Democratic Republic of Congo;; ¶ Hôpital Général de Kilo-Moto, Watsa, Democratic Republic of Congo

**Keywords:** Marburg hemorrhagic fever, filovirus epidemiology, secondary attack risk, Democratic Republic of Congo, research

## Abstract

The first major outbreak of Marburg hemorrhagic fever (MHF) outside a laboratory environment occurred in the subdistrict of Watsa, Democratic Republic of Congo, from October 1998 to August 2000. We performed a serosurvey of household contacts of MHF patients to identify undetected cases, ascertain the frequency of asymptomatic Marburg infection, and estimate secondary attack risk and postintervention reproduction number. Contacts were interviewed about their exposure and symptoms consistent with MHF. Blood samples were tested for anti–Marburg immunoglobulin G (IgG). One hundred twenty-one (51%) of 237 identified contacts participated; 72 (60%) were not known to the health authorities. Two participating contacts were seropositive and reported becoming ill after the contact; no serologic evidence for asymptomatic or mild Marburg infection was found. The secondary attack risk was 21%; the postintervention reproduction number was 0.9, consistent with an outbreak sustained by repeated primary transmission, rather than large-scale secondary transmission.

## Abstract 2

Asymptomatic secondary infections are rare.

Marburg hemorrhagic fever (MHF) is a rare disease caused by the Marburg filovirus; it occurs in central, east, and southern Africa. MHF is characterized by sudden onset of fever, headache, myalgia, arthralgia, and frequently progresses to diarrhea and vomiting, hemorrhagic diathesis (petechiae, hematemesis, melena), and death (*1*). Case fatality reached 88% in a community outbreak in Uige, Angola (*2*). No vaccine or antiviral therapy is available; supportive treatment consists primarily of correcting fluid and electrolyte imbalances. The putative diagnosis is established on clinical and epidemiologic grounds and confirmed by polymerase chain reaction (PCR), antigen-capture enzyme-linked immunosorbent assay (ELISA), immunoglobulin M (IgM) ELISA, or virus isolation.

The reservoir animal species capable of surviving Marburg infection and sustaining the virus's lifecycle has not been discovered (*3*); thus, transmission patterns from the reservoir to humans are not known. Transmission between humans occurs through direct contact with symptomatic MHF patients or with their body fluids or remains (*4*). The risk for transmission of Marburg virus is assumed to increase with the intensity of physical contact and the amount of body fluids shed, as shown for Ebola virus (*5*).

The first major community outbreak of MHF described (>150 putative cases, case fatality 83%) was in the mining village of Durba and the neighboring town, Watsa, in the northeast of the Democratic Republic of Congo (DRC), in 1999. The outbreak probably started in October 1998, had several peaks alternating with latent periods, and ended in August 2000, when the last confirmed MHF cases occurred (*6*). Primary cases were predominantly in *orpailleurs* (unofficial gold miners), while secondary cases were predominantly in household contacts and healthcare workers. Response activities similar to those for Ebola outbreaks were started in May 1999 with temporary assistance from expert teams. These measures included active and passive surveillance, follow-up of contacts, isolation of cases, barrier nursing, and safe burials (*7*).

The surveillance system likely did not identify all MHF cases because surveillance officers did not make sufficient efforts to approach families of primary case-patients, patients with mild cases were not referred to an experienced clinician for assessment (*8*), or contacts concealed symptoms compatible with MHF to avoid isolation. We carried out a serosurvey of household contacts to ascertain unidentified MHF cases and to estimate the secondary attack risk and postintervention reproduction number.

## Methods

### Study Area and Population

The epicenter of the 1998–2000 MHF outbreak, Durba, is 14 km from Watsa town, the administrative center of the subdistrict of Watsa. Watsa Subdistrict is located near the border with Uganda and Sudan. Watsa's health system was seriously compromised during the outbreak by economic decline and the ongoing war in eastern DRC.

Our survey was of lay persons, referred to as household contacts, whose contact with an MHF patient occurred during lay activities, such as nursing a patient (supporting, feeding, washing, and the like, whether at home or in health facilities), transporting a patient or body, or preparing a body for burial. Healthcare workers whose contact occurred during their professional duties were not eligible.

Cases of MHF were either laboratory confirmed (positive by PCR, antigen-capture ELISA, virus isolation, or a combination of IgG ELISA, IgG indirect immunofluorescence assay [IFA], and clinical and epidemiologic evidence [48 cases]) or epidemiologically linked (persons for whom laboratory confirmation was not attempted who had acute fever, hemorrhage, and contact with a laboratory-confirmed patient [25 cases]). Forty-five cases were known from surveillance during the outbreak; we identified 28 retrospectively. Contacts of suspected case-patients whose conditions were not laboratory confirmed or epidemiologically linked as defined above were not eligible because their diagnosis lacked certainty.

We attempted to visit the households of all 73 MHF patients and to prepare a list of persons who had direct contact with the patient or his or her body fluids or remains. If contacts were temporarily absent, we undertook at least 2 repeat visits. If they had moved away, we tried to locate them at their new address, unless distance or lack of security (e.g., rebel activity, bandits) hindered us, in which case we interviewed former neighbors about the contacts' disease episodes in the 4 weeks after the patient's illness. We asked all contacts we met to give verbal informed consent; if they agreed, we interviewed them and took blood samples. This study was approved by the ethics committee of the Antwerp Institute for Tropical Medicine and the representative of the Ministry of Health in Watsa.

### Interviews

After establishing the identity of the contact and the relationship to the patient, we asked an open-ended question about the role the contact played during the patient's illness. We also asked closed-ended questions on whether the contact had touched, carried, or embraced the patient (and whether the patient at that point had diarrhea, vomiting, or bleeding) and whether the contact had touched the patient's clothes or linen (and whether these were soiled with stool, vomitus, or blood). Since patients who died often had had diarrhea, vomiting, and bleeding in the final stages of disease, we also asked whether the contact had touched, carried, embraced, or washed the person after death. While field testing the questionnaire, we found that protective gear such as gloves was unavailable to lay persons; thus, all contacts were assumed to be unprotected. We asked about symptoms the contact had experienced during the 4 weeks after exposure; these symptoms (Table 1) correspond to the ones used during the epidemic to define a clinically suspected case.

**Table 1:** Symptoms in 121 household and community contacts within 4 weeks after exposure to a Marburg hemorrhagic fever patient, Watsa Subdistrict, Democratic Republic of Congo, 2002

Symptoms		No. seronegative (%), n = 119	No. seropositive (%), n = 2	Total (%), N = 121
Fever		37 (31.1)	1	38 (31.4)
General symptoms				
	Headache	55 (46.2)	1	56 (46.3)
	Fatigue	45 (37.8)	2	47 (38.8)
	Loss of appetite	39 (32.8)	1	40 (33.6)
	Joint pain	33 (27.7)	1	34 (28.1)
	Muscle pain	26 (21.9)	1	27 (22.3)
	Back pain	24 (20.2)	1	25 (20.7)
	Abdominal pain	23 (19.3)	2	25 (20.7)
	Chest pain	14 (11.8)	2	16 (13.2)
	Nausea, vomiting	10 (8.4)	2	12 (9.9)
	Diarrhea	11 (9.2)	1	12 (9.9)
	Dyspnea	8 (6.7)	2	10 (8.3)
	Sore throat	8 (6.7)	1	9 (7.4)
	Hiccough	3 (2.5)	2	5 (4.1)
	Any general symptom	68 (57.1)	2	70 (57.9)
Hemorrhage				
	Nose bleed	2 (1.7)	0	2 (1.7)
	Bloody/black stool	1 (0.8)	1	2 (1.7)
	Coughing blood	1 (0.8)	1	2 (1.7)
	Bloody vomit	0	1	1 (0.8)
	Vaginal bleeding	1 (0.8)	0	1 (0.8)
	Any hemorrhage	3 (2.5)	1	4 (3.3)
Combinations				
	Fever + >3 general symptoms	32 (26.9)	1	33 (27.3)
	Fever + hemorrhage	2 (1.7)	1	3 (2.5)
	Clinically suspect case*	32 (26.9)	1	33 (27.3)
Total		119 (100.0)	2	121 (100.0)

*Fever + >3 general symptoms or fever + hemorrhage.

### Blood Sampling and Testing

After the interview, 5–10 mL venous blood was taken from contacts. After 12 to 24 hours, serum was separated from the blood clot, refrigerated at ≈4°C, and transported to the Uganda Virus Research Institute (within 1 to 2 weeks). There it was frozen at –70°C and shipped on dry ice to the National Institute for Communicable Diseases, Johannesburg. Serum was examined by ELISA and IFA and considered positive if anti-Marburg IgG was found in both tests.

### Data Analysis

Data were entered with EpiInfo version 6.0 (Centers for Disease Control and Prevention, Atlanta, GA, USA), and analyzed with Stata version 8.2 software (StataCorp LP, College Station, TX, USA). Depending on symptoms associated with increased virus shedding and on the intensity of the contact, level of exposure was categorized. Low-level contact included any direct contact with a living case-patient without diarrhea, vomiting, or bleeding; or touching clothes or sheets not soiled with stool, vomitus, or blood. Medium-level contact was defined as touching a living case-patient with diarrhea, vomiting, or bleeding; touching clothes or sheets soiled with stool, vomitus, or blood; or touching remains. High-level contact included carrying or embracing a living patient who had diarrhea, vomiting, or bleeding; or carrying, embracing, or cleaning remains.

We established transmission chains and generations for all patients, taking into account work as a gold digger, exposure to other patients, incubation period, and date of onset (M. Borchert, unpub. data). When a patient had been working as a gold digger and had been exposed to another patient, we gave priority to the confirmed human-to-human exposure over the possible primary exposure and classified these cases as nonprimary ones.

We calculated the secondary attack risk as the proportion of household contacts of primary case-patients who then became secondary case-patients themselves, including only primary case-patients whose contact list could be established fully and who did not share contacts with another case-patient; we used the analogous approach to estimate the tertiary attack risk. We computed the reproduction number (*R_p_*) as the product of the secondary attack risk and the average number of contacts per primary case-patient. As most cases in our survey had occurred after control measures were implemented, we consider this number to be the postintervention *R_p_*, not the basic reproduction number *R*_0_.

## Results

### Completeness of Data

Household contacts of 73 MHF patients were eligible to participate in the survey. We completed contact lists for 48 patients (66%). For 7 patients, Watsa health authorities had listed some contacts during the epidemic. Because we could not meet these patients, their contacts, or others who could verify the list's completeness we likely missed some contacts. For 18 patients, no contacts had been listed by the health authorities. Since we did not speak to anyone who had witnessed the case during the epidemic, contacts have also probably been missed for these cases.

Existing surveillance records listed 141 contacts. For the 48 cases we could investigate fully, 96 additional contacts were found. Seventy-one of these were contacts of patients identified by surveillance during the outbreak. The total number of identified contacts therefore was 237 (141 + 96), relating to 55 (48 + 7) of 73 cases.

A patient whose case was fully investigated had, on average, 4.46 contacts; on this basis, one would expect 326 contacts for all 73 patients. The 237 identified contacts correspond to 73% of this expected number. A total of 143 contacts could be traced, and 124 consented to being interviewed and giving a blood sample, representing 52% of the 237 identified contacts and 38% of the 326 expected contacts. Three persons listed by surveillance denied any physical contact with the patient and were excluded from analysis. Therefore, results refer to 121 study participants.

### Characteristics of Contacts

The median interval between the onset of the patient's disease and the contact's interview and blood sample collection was 24 months (range 11–48). Half of the contacts were female, and three fourths were 15–49 years of age. Most contacts were family members (88%), while colleagues accounted for 11% (Table 2).

**Table 2:** Characteristics of 121 household or community contacts of Marburg hemorrhagic fever patients, Watsa Subdistrict, Democratic Republic of Congo, 2002

Characteristics		No. male (%), n = 63	No. female (%), n = 58	Total (%), N = 121
Age (y)				
	<4	2 (3.5)	0	2 (1.7)
	5–14	3 (5.2)	5 (7.9)	8 (6.6)
	15–29	24 (41.4)	35 (55.6)	59 (48.8)
	30–44	20 (34.5)	11 (17.5)	31 (25.6)
	>45	9 (15.5)	12 (19.1)	21 (17.4)
Residence				
	Durba	38 (65.5)	47 (74.6)	85 (70.3)
	Watsa town	9 (15.5)	7 (11.1)	16 (13.2)
	Other village in Watsa Health Zone	8 (13.8)	5 (7.9)	13 (10.7)
	Outside Watsa Health Zone	3 (5.2)	4 (6.4)	7 (5.8)
Profession*				
	Housewife	0	29 (46.0)	29 (24.0)
	Unofficial gold miner	23 (39.7)	0	23 (19.1)
	Pupil/student	6 (10.3)	9 (14.3)	15 (12.4)
	Farmer	7 (12.1)	7 (11.1)	14 (11.6)
	Trader	2 (3.5)	11 (17.5)	13 (10.7)
	Health worker	2 (3.5)	2 (3.2)	4 (3.3)
	Other or none	16 (27.6)	2 (3.2)	18 (14.9)
Relationship				
	Spouse	3 (5.2)	12 (19.5)	15 (12.4)
	Same generation as case (brother, sister, brother- or sister-in-law, cousin)	24 (41.4)	18 (28.6)	42 (34.7)
	Subsequent generation (son/daughter, nephew or niece)	13 (22.4)	13 (20.6)	26 (21.5)
	Preceding generation (father or mother, uncle or aunt)	7 (12.1)	16 (25.4)	23 (19.0)
	Colleague	10 (17.2)	3 (4.8)	13 (10.7)
	Other	1 (1.7)	1 (1.6)	2 (3.3)
Total		63 (100)	58 (100)	121 (100)

*N = 116 because of missing data.

Half of the contacts held or carried a patient, a third fed or washed a patient, and a tenth reported sharing a bed with a patient (Table 3). Exposure to a living patient was almost universal; three quarters of contacts had exposure to body fluids and excreta. Forty-three percent of contacts had exposure to remains. The exposure level was low in 13%, medium in 19%, and high in 68% of contacts and did not differ between the sexes.

**Table 3:** Type and level of exposure of 121 household and community contacts of Marburg hemorrhagic fever patients, Watsa Subdistrict, Democratic Republic of Congo, 2002

Exposure		No. male (%), n = 58	No. female (%), n = 63	Total (%), N = 121
Role played for the living patient				
	Held or carried	33 (56.9)	31 (49.2)	64 (52.9)
	Fed	14 (24.1)	24 (38.1)	38 (31.4)
	Washed	14 (24.1)	24 (38.1)	38 (31.4)
	Washed patient's clothes	4 (6.9)	19 (30.2)	23 (19.0)
	Made patient drink	6 (10.3)	16 (25.4)	22 (18.2)
	Shared bed	4 (6.9)	8 (12.7)	12 (9.9)
	Give medical care	6 (10.3)	5 (7.9)	11 (9.1)
Contact with living patient				
	Touched with hands	54 (93.1)	58 (92.1)	112 (92.6)
	Touched a patient with diarrhea, vomiting, bleeding	40 (69.0)	44 (69.8)	84 (69.4)
	Carried, embraced, or shared bed	42 (72.4)	44 (69.8)	86 (71.1)
	Carried, embraced or shared bed when patient had diarrhea, vomiting, bleeding	31 (53.5)	37 (58.7)	68 (56.2)
	Touched object like clothes or sheets with hand	33 (56.9)	43 (68.3)	76 (62.8)
	Touched objects when soiled with stool, vomit, blood	24 (41.4)	36 (57.1)	60 (49.6)
	Any physical contact with living patient or object	54 (93.1)	59 (93.7)	113 (93.4)
	Any physical contact with patient or object, with putative exposure to stool, vomit, blood	41 (70.7)	49 (77.8)	90 (74.4)
Contact with remains of patient				
	Touched with hands	27 (46.6)	25 (39.7)	52 (42.9)
	Carried or embraced	18 (31.0)	17 (27.0)	35 (38.9)
	Cleaned	9 (15.5)	7 (11.1)	16 (13.2)
	Any physical contact	27 (46.6)	25 (39.7)	52 (43.0)
Level of exposure				
	Low level of physical contact*	9 (15.5)	7 (11.1)	16 (13.2)
	Medium level of physical contact†	10 (17.2)	13 (20.6)	23 (19.0)
	High level of physical contact‡	39 (67.2)	43 (68.3)	82 (67.8)
Total		58 (100.0)	63 (100.0)	121 (100.0)

*Low, any direct contact with living patient who did not have diarrhea, vomiting, or bleeding; touching clothes or sheets not soiled with stool, vomit, or blood. †Medium, touching living patient who had diarrhea, vomiting, or bleeding; touching clothes or sheets soiled with stool, vomit, or blood; touching remains. ‡High, carrying or embracing living patient who had diarrhea, vomiting, or bleeding; carrying, embracing, or cleaning remains.

For 43 of the 50 contacts known to surveillance, we compared exposure reported in our survey with exposure documented by surveillance officers during the outbreak. For 88% of contacts, surveillance and study information agreed.

Two study participants were positive for anti–Marburg IgG: a 21-year-old brother and a 27-year-old male neighbor of MHF patients. Both contacts were highly exposed to their respective primary case-patients. These contacts were also, as unofficial gold miners, at risk for primary transmission themselves (*6*). The 21-year-old reported 6 general symptoms within 4 weeks after exposure, including fatigue, abdominal pain, nausea/vomiting, hiccoughs, chest pain, and difficulty breathing, but he did not fulfill the definition of a suspected case because he did not exhibit fever or bleeding. The 27-year-old reported a hemorrhagic fever syndrome, including vomiting and coughing blood and bloody or black stool. Neither contact sought medical care. We consider them to be additional confirmed patients and classified them as secondary cases because of the combination of high exposure and postexposure symptoms compatible with MHF. The 119 seronegative contacts were considered nonpatients. Thus, the overall seroprevalence in our study population is 1.65% (95% confidence interval [CI] 0.2%–5.8%), the same as in the general population (1.64%) (*6*).

Although almost all contacts were seronegative, one third reported fever within 4 weeks of contact with a patient (Table 1), more than one half reported a general symptom (headache, fatigue, and loss of appetite most frequently), and 3.3% reported hemorrhage. Thirty-three (27%) contacts would have qualified as clinically suspected case-patients during the epidemic and should have been taken to an isolation ward for assessment by an experienced healthcare worker. This did not happen, and 23 of these persons were not even known by authorities to be contacts.

On the basis of surveillance records and interviews with family members, neighbors, or colleagues of the 113 eligible contacts we could not interview or obtain blood samples from, we identified 1 epidemiologically linked patient, 1 suspected MHF case-patient, and 13 noncases; for the 98 remaining contacts, information was insufficient to classify them. The total of MHF cases thus increased to 76 (50 laboratory-confirmed, 26 epidemiologically linked).

### Secondary Attack Risk and Postintervention *R_p_*

Thirty-one of 76 cases were identified as primary, 21 as secondary, 15 as tertiary, and 5 as quaternary. Four cases could not be classified. Eleven patients with secondary cases acquired their infection as a household contact and had only 1 patient with a fully investigated primary case as a possible source. These constituted the numerator for the secondary attack risk and contributed to the denominator. Forty-two healthy contacts with only 1 patient with a fully investigated primary case as possible source also contributed to the denominator. The secondary attack risk was thus estimated as 21% (11/[11 + 42], 95% CI 11–34) for household contacts. Restricting the calculation to confirmed primary cases did not significantly change the secondary attack risk estimate. The average number of household contacts per fully investigated primary case was 4.46, so that *R_p_* for household contacts was estimated as 0.93. The tertiary attack risk (6/32 = 19%, CI 7–36) did not differ from the secondary one.

## Discussion

Most of the 121 household and community contacts of MHF patients reported substantial unprotected exposure to Marburg virus through physical contact with patients, their body fluids, or remains. In addition to the secondary cases identified through surveillance, we found serologic evidence for Marburg infection in 2 persons and epidemiologic evidence in 1 person. For all 3 persons, substantial clinical disease after the exposure was reported. As most patients identified during the Watsa outbreak showed signs of disease (D.G. Bausch et al., unpub. data), we conclude that mild or asymptomatic Marburg infection, albeit possible (*8*), was a rare event.

One fourth of the seronegative contacts reported symptoms within 4 weeks of exposure, which fulfilled the definition for a suspected case. This figure illustrates the difficulty in deciding whether to isolate patients on the basis of clinical and epidemiologic data alone. The risk for cross-contamination on the isolation ward, if persons are incorrectly hospitalized, and the risk for continued community transmission, if true cases are not isolated, show the necessity of having a laboratory diagnosis available within 1 or 2 days.

Our secondary attack risk estimate of 21% is within the range reported for Ebola outbreaks for comparable types of contacts: Ebola-Zaire, Kikwit, 1995, household contacts 16% (*5*); Yambuku, 1976, close relatives, 20% (*9*); Ebola-Sudan, Nzara, 1979, family members with physical contact including nursing 31% (*10*) Our estimate is much higher than the 2.5% reported for Ebola-Sudan, Uganda, 2000 (*11*); however, the Ugandan estimate may have included persons who merely stayed in the same house as a patient without reporting physical contact. The secondary and tertiary attack risks in our study were found to be virtually identical, 21% and 19%, respectively; thus no evidence suggested that Marburg virus loses infectivity by repeated passages through humans.

We found the postintervention reproduction number *R_p_* to be <1; after the implementation of control measures, secondary transmission was not sustainable in the community. This finding is consistent with our observations during the outbreak, whose prolonged duration of almost 16 months after control measures were initiated in May 1999 was due to repeated primary transmission into the human population and not to sustained secondary transmission. The outbreak ended when the dominant location of primary transmission, the Gorumbwa gold mine, ceased to be accessible (D.G. Bausch et al., unpub. data). Our data do not allow computing the preintervention basic reproduction number *R_0_*, so we cannot be certain how much of a difference the control measures made, but we think they had some effect.

The proportion of contacts (71/212) and the number of clinically suspected cases (33) missed by surveillance were high. Two of 3 retrospectively identified MHF patients were contacts of patients known to the health authorities. These contacts reported symptoms that qualified them as having suspected cases, but they were missed nevertheless. Given the importance of early recognition and isolation of MHF patients for outbreak control, this finding raises the question of how the Watsa health authorities could have been better supported in their surveillance activities. After Watsa's chief medical officer died from MHF in May 1999 (*12*), the post remained vacant for many months. We suggest that continuous support to the health zone by training and deploying a Congolese epidemiologist might have been more cost-effective than the intermittent support provided by experts from May 1999 to October 2000. This strategy would also have strengthened DRC's capacity to deal with future viral hemorrhagic fever outbreaks.

The survey's setting was characterized by high mobility because of the war and the flooding of the Gorumbwa gold mine, and we could not investigate all cases fully because of lack of available sources. For these reasons, we located at best half of all contacts and probably fewer. We could not make firm conclusions about those whom we could not interview or obtain blood from: a few may have contracted or even died from MHF. However, contacts for whom no information was available were no more likely to contract MHF than those we could study. In settings where families are isolated from their surroundings, a filovirus may wipe out a household, leaving no witness to report the event. In Durba and Watsa, where households are physically and socially close, such a tragedy is unlikely to have happened without anyone noticing, remembering, and reporting; we therefore believe that a substantial survival bias is unlikely.

The accuracy of reported exposure and symptoms may have had recall bias, given the average interval of 2 years between the patient's disease and the survey. When our data were compared with exposure information recorded by surveillance officers during the outbreak, agreement was satisfactory, however. Exposure patterns reflected traditional female and male roles in caring for diseased relatives. Since no material gains were offered to newly identified patients, we did not provide incentives to overreport exposure or symptoms. Giving a blood sample is unpopular in the study setting; to avoid underreporting exposure and symptoms, study participants were informed before the interview that a blood sample would be requested, regardless of their answers to interview questions. Those who did not wish to be interviewed or provide a blood sample refused overtly. In summary, we believe the interview data are valid.

If anti–Marburg IgG antibodies were transitory after infection with Marburg virus, they might have fallen below detectable levels in the interval between exposure and blood collection. However, samples taken from 17 MHF survivors after 22 to 102 months of follow-up that were stored, transported, and analyzed in the same way as the samples of this survey showed that none became seronegative. These persons from the 1994 or 1998–2000 MHF outbreaks became seropositive during or shortly after disease and included 2 with mild Marburg disease (M. Borchert, unpub. data). We conclude that Marburg antibodies persisted sufficiently to be detected in our serosurvey.

If some of our epidemiologically linked case-patients did not have MHF, this result could have diluted the secondary attack risk. However, restricting the analysis to confirmed cases did not increase, but rather reduced, the secondary attack risk, albeit not significantly. We report the secondary attack risks on the basis of confirmed and epidemiologically linked cases, which is equally valid and more stable because of the larger number of observations.

Calculation of secondary attack risk and *R_p_* depends on determining the transmission generations correctly. We are confident that our data are of sufficient quality to allow this, but an inherent uncertainty exists regarding patients who worked as unofficial gold miners and reported substantial exposure to another patient. We think these persons could be classified as having secondary cases, given the confirmed secondary, but uncertain primary, exposure. In the unlikely event that these were all primary cases, the secondary attack risk would be reduced to 16%, which would not change our conclusions substantially.

## Conclusion

We found that asymptomatic or very mild Marburg infection was a rare event in the Watsa outbreak. The postintervention reproduction number *R_p_* was <1, which suggests that the MHF outbreak in Watsa and Durba was sustained through repeated introduction of the virus into the human population and not through secondary spread. We showed that the identification and follow-up of contacts during the outbreak were incomplete and raised the question of how support for surveillance efforts in a health zone such as Watsa could be improved.
